# Is passion contagious in coach-athlete dyads? A dyadic exploration of the association between passion, affective and need-based experiences in individual sports

**DOI:** 10.3389/fpsyg.2024.1369011

**Published:** 2024-04-24

**Authors:** Marieke Fonteyn, Leen Haerens, Maarten Vansteenkiste, Tom Loeys

**Affiliations:** ^1^Department of Data Analysis, Ghent University, Ghent, Belgium; ^2^Department of Movement and Sports Sciences, Ghent University, Ghent, Belgium; ^3^Department of Developmental, Personality and Social Psychology, Ghent University, Ghent, Belgium

**Keywords:** passion, need satisfaction, positive affect, actor-partner interdependence model, bidirectional influence

## Abstract

The Dualistic Model of Passion distinguishes between harmonious and obsessive passion, which are associated with athletes’ and coaches’ adjustments. Whereas prior research sampled either athletes or coaches, the present study used a dyadic approach to explore the bidirectional influence of passion on affective experiences in coaches and athletes. Using a cross-sectional dyadic design, 198 coach-athlete dyads involved in an individual sport at different competition levels, reported on their passion, need-based, and affective experiences. Both actor effects (i.e., intrapersonal dynamics within athletes or coaches) and partner effects (i.e., interpersonal dynamics from coach to athlete and vice versa) were examined. Furthermore, dyadic mediation models were used to investigate the potential mediating role of need-based experiences in the association between passion and affective experiences. Results unveiled compelling evidence for actor effects, indicating that one’s own harmonious passion was positively related to one’s own more adaptive outcomes and negatively to one’s own more maladaptive outcomes, whereas obsessive passion was positively related to maladaptive outcomes. Further, very limited evidence for partner effects, in which coaches’ passion affected athletes’ outcomes or vice versa, was found. The dyadic mediation models underscored the role of need-based experiences in mediating the association between passion and affective experiences, but only at the intrapersonal level. As such, one’s own passion experiences were related to one’s own need-based experiences, which in turn were related to one’s own affective experiences. The study provided no evidence for interpersonal mediation effects.

## Introduction

“As coaches, this is our dream also. It’s my career, it’s my passion and it has been great being on this journey with her.” (Aimee Boorman, Simone Biles’ former coach).

The role of passion in sports has been researched as a crucial factor in shaping athletes’ sport experiences, including their emotional experiences (e.g., Vallerand et al., 2006; Verner-Filion et al., 2014; Stenseng et al., 2015), cognitive experiences (e.g., Philippe et al., 2009), burn-out perceptions and experiences (e.g., Gustafsson et al., 2011; Curran et al., 2013; Martin and Horn, 2013; Kent et al., 2018; Lopes and Vallerand, 2020), risk behaviors and sport dependence (e.g., Akehurst and Oliver, 2014). Yet, Aimee Boorman’s quote (St. Clair, 2016) demonstrates that such experiences of passion are not confined solely to athletes. Coaches too can harbor profound passion for sports, which they potentially transmit to their athletes.

Over the past years, some studies already have focused on coaches’ passion (e.g., Kim et al., 2019; Yukhymenko-Lescroart and Sharma, 2022), complementing the extensive research focus on athletes’ passion (Vallerand et al., 2003; Vallerand, 2015; Vallerand and Verner-Filion, 2019). Yet, most of these studies examined passion from a unidirectional perspective, in which athletes or coaches were studied in isolation. Hence, one of the understudied questions in the current passion literature pertains to the transmission effects of coaches’ (athletes’) passion on athletes’ (coaches’) adjustment, and whether the manifestation of this contagion effect depends on the type of passion (i.e., harmonious versus obsessive).

To address these questions, it is both necessary and essential to adopt a dyadic perspective and to acknowledge the bidirectional nature of the coach-athlete relationship. This implies that the interdependence between coaches and athletes is taken into account by considering the experiences and characteristics of both athletes and coaches. Such an approach enables the concurrent examination of both actor (i.e., intrapersonal dynamics within coaches and athletes) and partner (i.e., interpersonal dynamics from coach to athlete and vice versa) effects, thus shedding a more accurate and richer light on the complexity of dynamics in the coach-athlete relationship (Kenny et al., 2006; Fonteyn et al., 2022). Specifically, this approach allows investigating whether the well-documented effect of passion on one’s own functioning holds when controlling for the dyadic nature of the coach-athlete relationship and, second, whether a transfer can be observed from either coaches’ or athletes’ passion to the change of the athlete or coach.

### The dualistic model of passion

The Dualistic Model of Passion (DMP; Vallerand et al., 2003; Vallerand, 2015), which is grounded in Self-Determination Theory (Ryan and Deci, 2017; Ryan, 2023), offers a comprehensive framework for understanding the intricate nature of passion across various domains of life. According to the DMP, passion refers to a strong inclination toward a self-defining activity or object that is highly valued, loved and to which the passionate person devotes a significant amount of time and energy on a regular basis (Vallerand, 2015). Yet, not all passions are created equal. Although individuals with either high harmonious or obsessive passion are intrinsically motivated for their activity, their intrinsic motivation gets coupled with additional motivations that differ in their level of internalization. In the case of harmonious passion (HP), the passionate activity is well-anchored within people’s lives, being balanced with other valued activities or life domains. Because the passionate activity is well-integrated, individuals freely and volitionally engage in their passions, thereby experiencing little conflict with other domains of their lives. Take the hypothetical example of Anne. Anne is a youth tennis coach. She is passionate about tennis and invests an incredible amount of time and energy into coaching. Beyond her love for tennis, Anne also cherishes her role as a parent, enjoys fine food and deems it essential to devote time to other hobby’s such as mountain biking and running. Coaching tennis players aligns harmoniously with these other important aspects of her life.

In the case of obsessive passion (OP), the (successful) engagement in the passionate activity has direct implications for individuals’ self-worth. Individuals with high obsessive passion experience a strong urge to exercise their passionate activity to preserve their self-worth and avoid feelings of guilt or anxiety. Introjected motives thus push the person into the passionate activity, such that obsessively passionate persons may experience more difficulties in finding resonance between their passionate activity and other areas of life and other parts of their identity (Vallerand et al., 2003; Vallerand, 2015; Vallerand and Verner-Filion, 2019). Consider Patrick as an example. Patrick is a swimming coach who dedicates himself to his role as a coach. Swimming holds utmost significance in his life. His passion is driven by his personal situation, preferences, and feelings, resulting in a diminished focus on the experiences of others. Apart from his role as a swimming coach, he is also a father of two children. His insatiable drive to immerse himself in swimming leads to conflicts of interest, notably within the domain of his family life.

Consistent with the idea that both passions are different, numerous unidirectional studies have shown that HP is positively associated with more favorable and adaptive outcomes such as attention and concentration (e.g., Vallerand et al., 2003), well-being, enjoyment and satisfaction with life (e.g., Schellenberg et al., 2021), whereas OP is less strongly or not related to these adaptive outcomes and sometimes even predicts maladaptive consequences such as injury likelihood (Schellenberg et al., 2021), burnout (Gustafsson et al., 2011; Martin and Horn, 2013; Moen et al., 2018), and stress and worry (Schellenberg et al., 2021). On the other hand, high levels of HP appear to function as a protective mechanism against maladaptive processes and outcomes including experiencing concentration problems (Philippe et al., 2009), and burnout (Martin and Horn, 2013; Moen et al., 2018). A similar pattern emerges when considering affective experiences [for an overview, see Curran et al. (2015)]. Research has explored the association between passion and indicators of general affect, as well as between passion and more specific affective experiences during and after activity engagement, both within and beyond the domain of sports. The majority of these studies yield consistent findings, namely that individuals with HP tend to report higher levels of positive affect and lower levels of negative affect. Conversely, OP is associated with higher levels of negative affect, while the association with positive affect is often weaker, negative or non-existing (Vallerand et al., 2006; Philippe et al., 2009; Stenseng et al., 2015; Moen et al., 2018; Schellenberg et al., 2021).

Also, when delving into more proximal variables in relation to passion, a discernible pattern emerges for harmonious and obsessive passion. As prior research has already identified need-based experiences as a proximal outcome that has been linked to passion in sports before (Verner-Filion et al., 2017; Lopes and Vallerand, 2020; Schellenberg et al., 2021), this study focuses on need satisfaction (NS) and need frustration (NF). Drawing form self-determination theory (SDT; Deci and Ryan, 2000; Ryan, 2023), it is hypothesized that individuals engage in activities to fulfill their basic psychological needs of autonomy (i.e., the need to experience personal initiative and to make volitional decisions), relatedness (i.e., the need for close, meaningful and reciprocal connections with others) and competence (i.e., the need to master skills that enable effective interactions with the environment). It has been argued that the satisfaction of these three needs contributes to individuals’ optimal functioning and personal growth, while the frustration of these needs can lead to impaired psychological growth, psychopathology and ill-being (Bartholomew et al., 2011; Vansteenkiste and Ryan, 2013).

In previous studies the role of these need-based experiences has been explored both as an outcome of passion (Schellenberg et al., 2021) in an athlete sample, and as mediator in the relationship between passion and more distal outcomes such as life satisfaction, deliberate practice and performance in hockey players (Verner-Filion et al., 2017), positive affective experiences in adult athletes from different recreational sports (Stenseng et al., 2015 for the mediation of the need to belong in the relation between passion and positive affect) and burnout in academy soccer players (Curran et al., 2013), in competitive athletes from different sport disciplines (Kent et al., 2018 for the mediation of autonomy satisfaction in the relation between passion and burnout) and in former or current athletes who engaged in running, triathlon, cycling, swimming or another sport (Lopes and Vallerand, 2020). When it comes to need satisfaction, these studies generally show a positive association between HP and need satisfaction, while the pattern for OP is less clear (e.g., Verner-Filion et al., 2017; Lopes and Vallerand, 2020). In some studies, no association between OP and need satisfaction was found (Stenseng et al., 2015; Verner-Filion et al., 2017; Schellenberg et al., 2021), while in other studies a positive, but weaker association (Lopes and Vallerand, 2020), or even a negative association was found (Tóth-Király et al., 2019; Schellenberg et al., 2021).

Less attention has been devoted to examining the relation between passion and experiences of need frustration. However, a study conducted by Schellenberg et al. (2021) investigated within-person combinations of HP and OP in relation to a broad range of outcomes in an athlete sample. One of the outcomes of interest was the athlete’s experiences of need thwarting. In this study the three needs (i.e., the need for autonomy, relatedness, and competence) were examined separately. The study’s results revealed a negative association between HP and the thwarting of each of the three needs, whereas for OP a positive association was found with need thwarting.

### Dyadic perspective

Up to this point, only unidirectional studies have been discussed, emphasizing the prevailing focus of passion studies on solely athletes’ or coaches’ experiences, with an implicit assumption that only actor effects were at play. This approach overlooks crucial interpersonal dynamics. Let us revisit the hypothetical scenario’s involving Anne and Patrick. Consider Anne, for instance. She not only acknowledges the significance of diverse domains and pursuits in her own life, but she also recognizes their value in the lives of her athletes. Therefore, she endeavors to foster this sense of balance in their athletic journey. As such, Anne’s focus on support is not exclusive to herself, but it extends to her athletes as well, resulting in feelings of connection with her athletes. In contrast, Patrick might adopt a different approach. For Patrick, swimming is one of the few things that truly matter. The swimmers under his guidance are affected by Patrick’s approach. One of Patrick’s swimmers, Amir, experiences several drawbacks stemming from Patrick’s passion. His unwavering focus on swimming leads to heightened expectations and even induces pressure on the swimmers to perform. Consequently, Amir not only feels nervousness but also a diminished sense of connection with other important aspects of his life and with his coach as their conversations revolve solely around swimming and performing. These underlying dynamics come to light only when adopting a dyadic perspective.

To the best of our knowledge, only very few studies studying the role of passion have adopted a dyadic perspective, thereby investigating both coaches and athletes. Fonteyn and Loeys (2022) investigated the impact of the COVID-19 pandemic on the association between passion and need satisfaction and frustration and between passion and affective experiences. One of the most interesting findings pertains to the relationship between passion and NF, where somewhat different results were observed compared to the study discussed above by Schellenberg et al. (2021). In the dyadic study conducted by Fonteyn and Loeys (2022) no association was found between OP and NF at both the intrapersonal and interpersonal level in coaches and athletes. The results regarding the association between HP and NF were more varied. Here, for athletes of coach-athlete dyads that were not impacted by the pandemic, HP was negatively related to their own NF experiences, while for coaches of coach-athlete dyads that were impacted by the pandemic, HP was negatively associated with both their own NF experiences and their athletes’ NF experiences. These results highlight how coaches’ HP may act as a protective factor against maladaptive outcomes for both themselves and their athletes. By adopting a dyadic approach, preliminary evidence into the simultaneous operation of both intrapersonal and interpersonal processes emerged, but it should be acknowledged that the sample size in this study was relatively small.

Further, Jowett et al. (2013) examined the role of passion in relation to more relational outcomes, namely relationship satisfaction and interpersonal conflict. Their study provided evidence for both actor and partner effects, resulting in a more fine-grained understanding of both intrapersonal and interpersonal passion dynamics within coach-athlete dyads across different sports.

The necessity of adopting a dyadic perspective when investigating dynamics within the coach-athlete relationship was also recently emphasized by Fonteyn et al. (2022). Ignoring the dyadic nature of the coach-athlete relationship by *a priori* assuming that only actor (i.e., intrapersonal) effects are at play, as is the case from the unidirectional perspective, may compromise an accurate representation and interpretation of real-life dynamics between coaches and athletes when also partner effects (i.e., interpersonal) are at play (Kenny et al., 2006; Fonteyn et al., 2022). Consequently, such flawed representation may also affect interventions in sport psychology practices, making them less effective.

**Figure 1:**
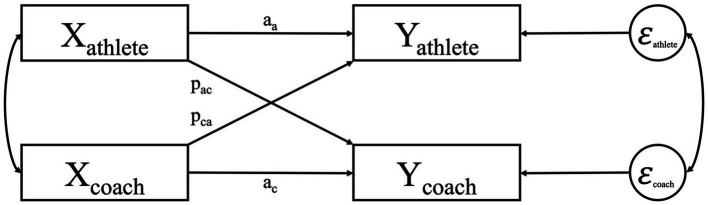
The actor-partner interdependence model for coach-athlete dyads. In the APIM, the horizontal arrows represent the actor effects (a) and the diagonal arrows represent partner effects (p). The correlation between the error terms at the right side of the figure reflects the residual non-independence in the outcome scores that is not explained by the model. The covariance between the coaches’ and athletes’ predictor variables is represented by the left double headed arrow.

One of the dyadic models that is often used to investigate bidirectional relationships is the Actor-Partner Interdependence Model (APIM; Figure 1; Kenny, 1996, 2018; Kenny and Cook, 1999; Kenny et al., 2006). The APIM is a dyadic model that allows to simultaneously estimate actor effects (i.e., intrapersonal effects or the effect of one’s own predictor variable on one’s own outcome variable) and partner effects (i.e., interpersonal effects or the effect of one’s own predictor variable on one’s partner outcome variable), while controlling for the other effect for both athletes and coaches. Further, the APIM also allows revealing dyadic patterns in the coach-athlete relationship. Typically, four patterns (i.e., configurations of actor relative to partner effects) are distinguished: the actor only pattern (i.e., the actor effect is different from zero and the partner effect is non-existing), the partner only pattern (i.e., the partner effect is different from zero and the actor effect is non-existing), the couple-oriented pattern (i.e., the actor and partner effects are equal) and the social comparison or contrast pattern (i.e., the actor and partner effects are equal in absolute magnitude, but opposite in sign) (Kenny and Cook, 1999; Kenny and Ledermann, 2010; Kenny, 2018).

### The current study

The current study aimed to address several goals. Firstly, we wanted to verify the robustness of the findings of prior unidirectional studies that have explored the association between passion and proximal need-based experiences and distal affective experiences. Therefore, this study used a dyadic perspective in which not only actor effects, but also partner transfer effects were acknowledged. By also investigating these transfer effects, new and unique insights may arise in how coaches’ passion may affect athletes’ sports experiences and vice versa. As illustrated in the hypothetical examples of Anne and Patrick, it is not implausible that the type of passion one experiences may also influence the individuals with whom they share close connections, in this case, the athletes collaborating with Anne and Patrick. The dynamics that may emerge in such situations could indeed be contingent upon the type of passion. While prior passion research that used such a dyadic approach focused on the association between passion and more relational outcomes, namely relationship quality and interpersonal conflict (Jowett et al., 2013) or focused on the impact of the COVID-19 pandemic on passion in coach-athlete dyads in a small sample (Fonteyn and Loeys, 2022), this study investigated the association between passion and more personal proximal (i.e., need-based experiences) and distal outcomes (i.e., affective experiences) in a large sample. As both more proximal and more distal variables were investigated in the study, mediation effects of the proximal processes in the relation between passion and the distal outcomes were also explored. To conclude, this study fills some important gaps in the current passion research literature. First, by utilizing a dyadic approach intrapersonal and transfer effects can be investigated. Second, we account for the potential mediation of proximal variables in the relation between passion and distal passion outcomes.

In line with prior research (e.g., Vallerand et al., 2003; Gustafsson et al., 2011; Curran et al., 2015; Fonteyn and Loeys, 2022), positive actor effects between harmonious passion and adaptive outcomes (i.e., positive affect and need satisfaction) and negative actor effects between harmonious passion and maladaptive outcomes (i.e., negative affect and need frustration) for both athletes and coaches were hypothesized. Further, positive actor effects between obsessive passion and maladaptive outcomes (i.e., negative affect and need frustration) for both athletes and coaches were expected. Given that prior studies have yielded mixed results regarding the association between obsessive passion and adaptive outcomes, we opt not to postulate an explicit direction for this relationship. Nonetheless, the association between OP and adaptive outcomes (i.e., positive affect and need satisfaction) was included in this study. The study also aims to examine the presence of partner effects. Given that partner effects have rarely been explored in the context of passion, we refrain from positing specific hypotheses and regard them as exploratory in nature. Finally, we investigated whether the proximal variables mediated the association between passion and the distal affective passion outcomes. By including mediation in the APIM both intrapersonal mediation effects (e.g., the effect of athletes’ need satisfaction in the association between athletes’ HP and athletes’ PA) as well as interpersonal mediation effects (e.g., the effect of athletes’ need satisfaction in the relationship between coaches’ HP and athletes’ PA) can be explored. This offers the opportunity to gain a comprehensive understanding of the underlying processes and transfer-effects that contribute to the relationship between passion and more distal affective experiences.

## Methods

### Participants

A total of 198 coach-athlete dyads from individual sports disciplines such as swimming, tennis, athletics, and gymnastics participated. These dyads were recruited from September 2020 until April 2021 across various competition levels, ranging from the recreational to the highly competitive level. The participants were required to be at least 16 years old to be eligible for inclusion in the study. The detailed demographic information of the sample is presented in Table 1.

**Table 1:** Demographic characteristics of coach-athlete dyads.

Baseline characteristics	*n* = 198
Athletes	
Age (years)	
Mean (*SD*)	24.57 (10.82)
Range	16–68
Competition level (*n*)	
High competitive	63 (31.82%)
Competitive	61 (30.81%)
Low competitive	24 (12.12%)
Recreational	50 (25.25%)
Sex (*n*)	
Male	80 (40.40%)
Female	117 (59.09%)
Other	1 (0.51%)
Experience (years)	
Mean (*SD*)	10.24 (6.24)
Range	0.5–40
Coaches	
Age (years)	
Mean (*SD*)	36.08 (13.16)
Range	18–72
Trainer degree (*n*)	
Yes	160 (80.81%)
No	38 (19.19%)
Sex	
Male	124 (62.63%)
Female	73 (36.87%)
Other	1 (0.51%)
Experience in coaching (years)	
Mean (*SD*)	12.51 (10.79)
Range	1–52
Hours of coaching per week (hours)	
Mean (*SD*)	13.76 (11.66)
Range	1–55
Coach-athlete relationship	
Years of collaboration (years)	
Mean (*SD*)	4.15 (3.43)
Range	0–18
Total hours of contact (hours)	
Mean (*SD*)	6.36 (8.82)
Range	0–100
One-to-one contact with coach (hours)	
Mean (*SD*)	1.30 (5.93)
Range	0–80

### Procedure

During the recruitment period, participants were sourced through various channels, including personal contacts of the involved researchers and master students, as well as posters and social media advertisement. Interested dyads were provided with information about the study’s objectives and procedures. Subsequently, an active informed consent was obtained from both athletes and coaches before participation. In the case of underage participants (age between 16 and 18 years old), a passive informed consent was asked from the participant’s parent(s) or legal guardian(s). Following consent, athletes and coaches were provided with a personalized web-link to complete an online survey, which approximately took between 20 and 30 min to complete. To ensure maximum participation, participants who did not respond to the initial invitation within a week, were send up to five reminder e-mails with a one-week interval. This research project was conducted in accordance with the ethical guidelines of the General and Specific Ethical Protocol of the Faculty of Psychology and Educational Sciences of Ghent University and has received approval from the ethical committee of this faculty.

### Measures

#### Passion for sport and coaching

To measure passion for sport and coaching in athletes and coaches, respectively, a Dutch-translated version of the Passion Scale (Vallerand et al., 2003) was utilized. The back translation procedure was used (International Test Commission, 2017) to ensure the accuracy of the translation process. Firstly, a researcher fluent in Dutch and familiar with sport psychology translated the original English instrument into Dutch. Secondly, an independent third person (a person with a master’s degree in languages) performed the back translations. Finally, a third person who was fluent in Dutch and English and familiar with the sports context, examined and compared the original and back translated items to ensure their equivalence. If there were any discrepancies, the researchers discussed and resolved them until they reached a consensus. The Passion Scale includes two six-items scales evaluating harmonious (e.g., “Doing sport is well integrated in my life”) and obsessive passion (e.g., “Coaching is so exciting that I sometimes lose control over it”). All items were rated on a seven-point Likert scale, ranging from 1 (“I do not agree at all”) to 7 (“I strongly agree”). For both scales a composite score was calculated by taking the mean for coaches and athletes. The internal consistencies of the scale were α_HP_ = 0.63 and α_OP_ = 0.82 for athletes and α_HP_ = 0.68 and α_OP_ = 0.82 for coaches.

#### Positive and negative affective experiences

To assess the affective experiences of coaches and athletes while coaching or participating in sports, respectively, the Positive and Negative Affect Schedule (PANAS; Watson et al., 1988) was used. A Dutch translation of the original scale was employed in this study, which was previously validated (Engelen et al., 2006). The PANAS comprises two scales that differentiate positive affect (PA) and negative affect (NA). For this study, only 10 items that were applicable and relevant to sports were selected from the original 20 item version. Each item reflects an emotional experience, with five items used to assess PA (e.g., enthusiastic) and five items to assess NA (e.g., nervous). Coaches and athletes were asked to rate each of these emotions while thinking about coaching or doing sports, respectively. The items were evaluated on a five-point Likert scale ranging from 1 (“Very slightly or not at all”) to 5 (“Extremely”). A sum-score was computed for both scales for coaches and athletes. The internal consistencies for athletes were *α*_PA_ = 0.75 and *α*_NA_ = 0.72. The internal consistencies for coaches were *α*_PA_ = 0.79 and *α*_NA_ = 0.60.

#### Basic psychological need satisfaction and frustration

Coaches’ and athletes’ basic psychological need satisfaction and frustration were evaluated using an adapted version of the Basic Psychological Need Satisfaction and Frustration Scale (BPNSFS; Chen et al., 2015). The sport-specific questionnaires for athletes and coaches (Delrue et al., 2019; Reynders et al., 2019) were modified to focus more on the coach–athlete relationship. For both need satisfaction and need frustration two items were used per need, resulting in six items for need satisfaction (e.g., “While practicing sports, I felt connected with my coach”) and six items for need frustration (e.g., “I feel I have no other choice but to coach athletes’). Respondents used a seven-point Likert scale ranging from 1 (“Totally not agree”) to 7 (“Totally agree”) to rate all 12 items. Composite scores for need satisfaction and need frustration were calculated for both athletes and coaches by taking the mean. The internal consistencies for athletes were *α*_NS_ = 0.69 and *α*_NF_ = 0.47. The internal consistencies for coaches were *α*_NS_ = 0.73 and *α*_NF_ = 0.61.

### Analysis plan

**Figure 2:**
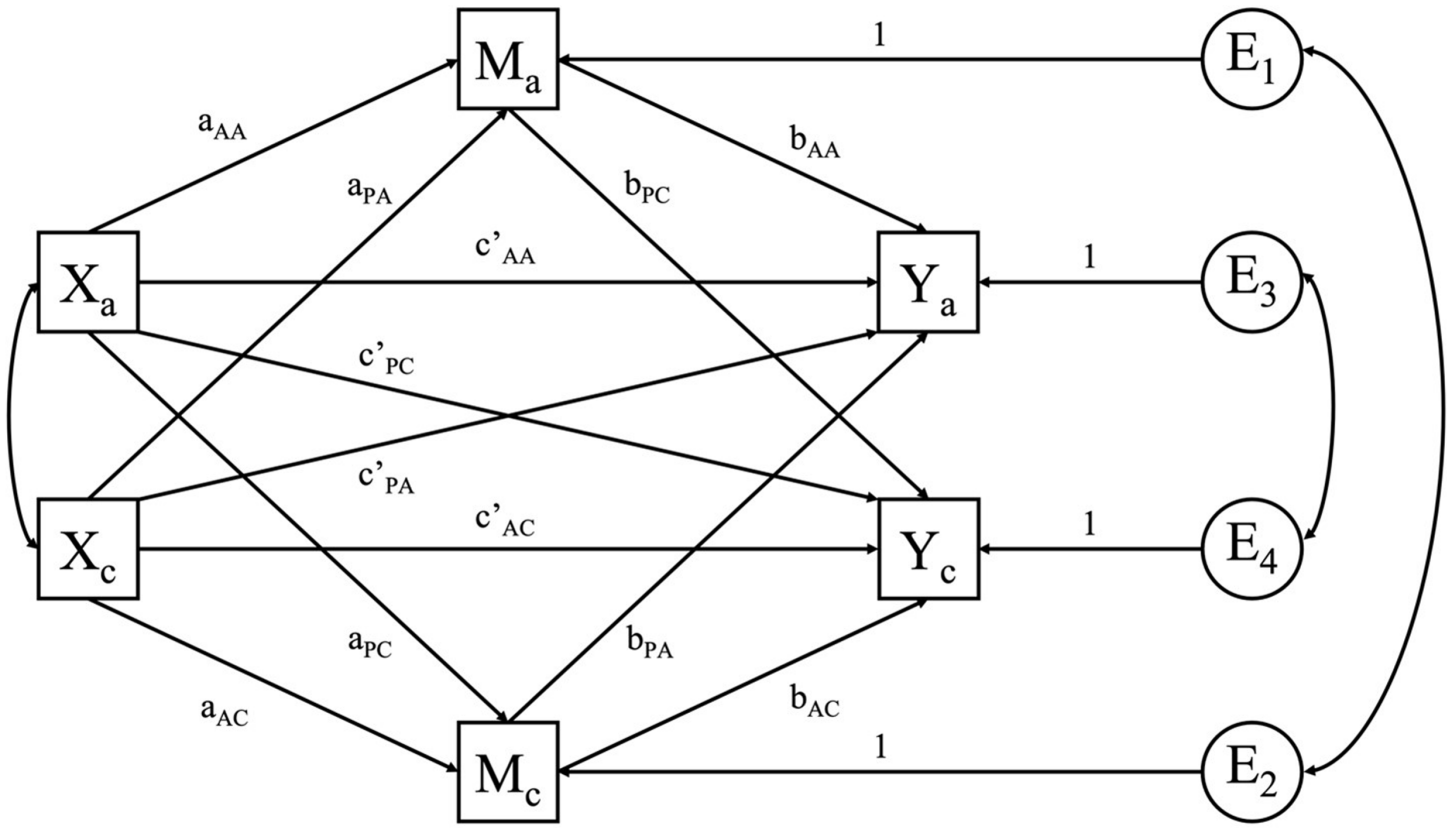
The actor-partner interdependence mediation model. In the Actor-Partner Interdependence Mediation Model, the *X* variables represent the predictor variables, the *M* variables represent the mediator variables, and the *Y* variables represent the outcome variables. For the second index, *a* refers to the athlete and *c* to the coach. The effect from *X* on *Y* is represented by the coefficient *c’*, the effect from *X* on *M* is represented by the coefficient *a* and the effect from *M* on *Y* is represented by the coefficient *b*. The first index refers to actor (a) or partner (p) effects.

The APIMs were fitted using Structural Equation Modeling through the R-package lavaan (Rosseel, 2012). Since we have two predictor variables (HP and OP) and four outcome variables (PA, NA, NS, and NF), eight APIMs (i.e., one for each combination) were fitted in total. To explore the potential mediation effect of the need-based experiences in the relationship between the two types of passion and the affective experiences, four Actor-Partner Interdependence Mediation Models (APIMeM; Figure 2; Ledermann et al., 2011) were fitted. In this model, the standard APIM is extended by a third pair of variables, the mediator variables. As such, in this model there are three pairs of variables, each measured in coaches and athletes: the two predictor variables (*X*_A_ and *X*_C_), the two mediator variables (*M*_A_ and *M*_C_) and the two outcome variables (*Y*_A_ and *Y*_C_). In the APIMeM, each mediator variable can mediate four relationships. For example, in the relationship between HP and PA with NS as a potential mediator, athletes’ NS is investigated as a mediator variable in the relation between athletes’ HP and athletes’ PA, coaches’ HP and coaches’ PA, athletes’ HP and coaches’ PA and coaches’ HP and athletes’ PA. In addition, in the APIMeM, each effect (i.e., the athlete actor effect, the coach actor effect, the athlete partner effect and the coach partner effect) consists of a direct effect and an indirect effect. The direct athlete actor affect for example is represented in Figure 2 by the arrow from *X*_A_ to *Y*_A_. The indirect athlete actor effect consists of the sum of the actor-actor simple indirect effect (i.e., the effect from *X*_A_ to *Y*_A_ via *M*_A_ in Figure 2) and the partner-partner simple indirect effect (i.e., the effect from *X*_A_ to *Y*_A_ via *M*_C_ in Figure 2). An overview of all the effects, the corresponding coefficients, and labels can be found in the Supplementary Table S2. Of note, while in prior research medium to high correlations were found between HP and OP (e.g., Vallerand et al., 2006, 2008; Lafrenière et al., 2008, 2011; Mageau et al., 2009; Jowett et al., 2013; Verner-Filion et al., 2014, 2017), these correlations were rather small in our sample (see Results section). As a consequence, separate models with HP and OP as single predictors were fitted to keep models more parsimonious. In this study, all tests were performed at the 5% significance level. No multiplicity correction was made. The sample size was pre-specified such that the study had 80% power to detect moderate actor and partner effects.

## Results

### Descriptive statistics

Baseline demographic information is presented in Table 1 and descriptive statistics and bivariate correlations among the study variables can be found in Table 2. As can be seen in Table 2, the Pearson’s bivariate correlations between coaches’ and athletes’ harmonious passion [*r* = 0.145, *t*(196) = 2.050, *p* = 0.042], obsessive passion [*r* = 0.242, *t*(196) = 3.495, *p* < 0.001], and negative affect [*r* = 0.279, *t*(196) = 4.060, *p* < 0.001] were moderate.

**Table 2:** Descriptive statistics and bivariate correlations of the coaches’ and athletes’ variables.

Study variables	1	2	3	4	5	6
1. Harmonious passion	0.145^*^	−0.065	0.452^***^	−0.098	0.468^***^	−0.211^**^
2. Obsessive passion	0.156^*^	0.242^***^	0.057	0.263^***^	0.018	0.282^***^
3. Positive affect	0.426^***^	0.246^***^	0.057	0.015	0.605^***^	−0.284^***^
4. Negative affect	−0.214^**^	0.259^***^	−0.249^***^	0.279^***^	−0.025	0.286^***^
5. Need satisfaction	0.424^***^	0.145^*^	0.336^***^	−0.252^***^	0.076	−0.352^***^
6. Need frustration	−0.366^***^	0.029	−0.432^***^	0.376^***^	−0.545^***^	0.104
*M*_coaches_	5.537	2.860	21.354	7.591	5.888	2.130
*SD*_coaches_	0.706	1.125	2.197	2.295	0.574	0.686
*M*_athletes_	5.641	3.585	20.707	8.707	5.858	2.108
*SD*_athletes_	0.682	1.229	2.469	2.773	0.626	0.612

Above the diagonal, the coaches’ bivariate correlations are presented. Below the diagonal, the athletes’ bivariate correlations are presented. On the diagonal, the bivariate correlations between the athletes’ and coaches’ variables are presented. ^***^p < 0.001, ^**^p < 0.01, ^*^p < 0.05.

### Actor-partner interdependence models

**Figure 3:**
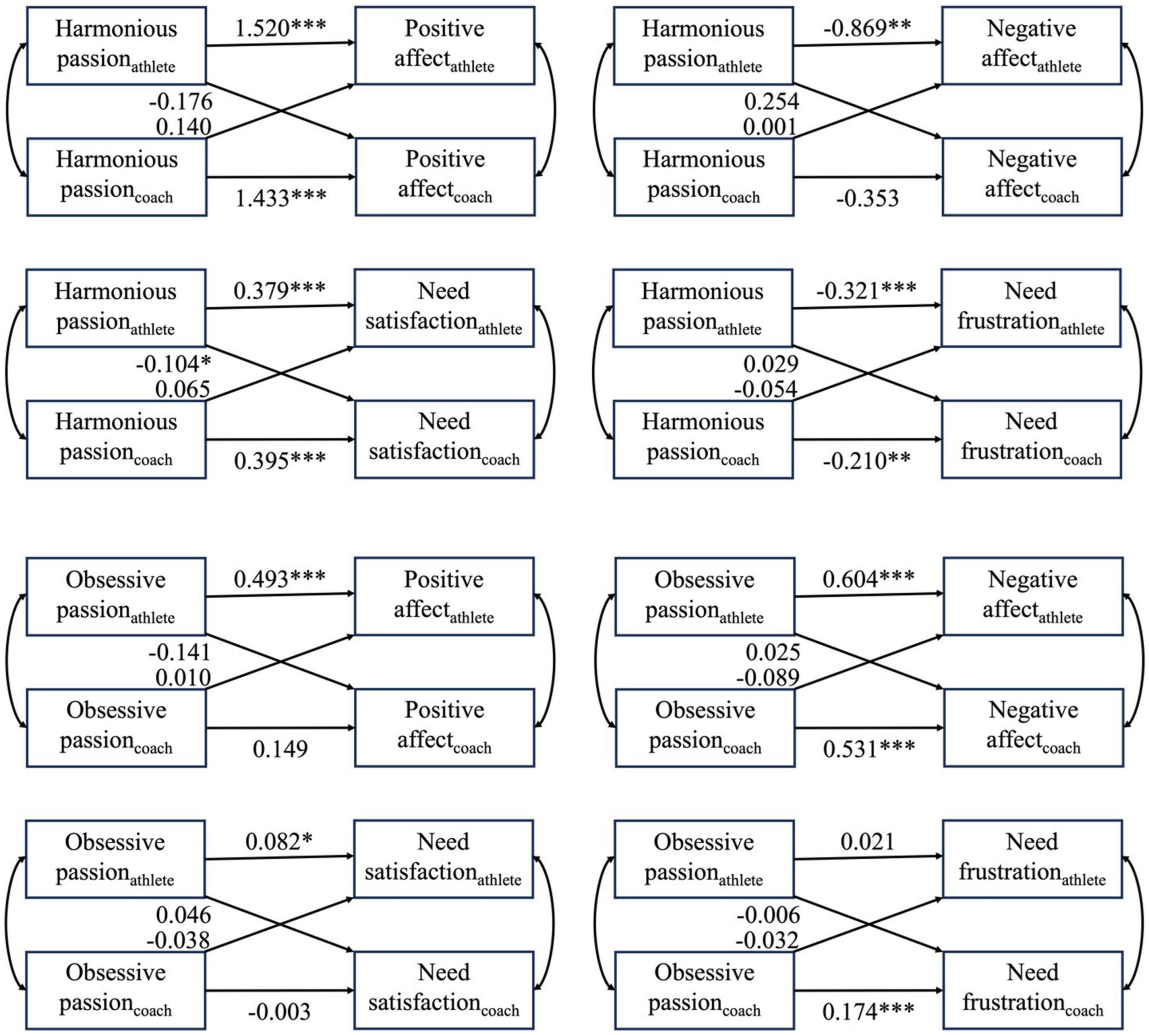
A visual representation of the estimated actor and partner effects for all the actor-partner interdependence models. The estimates of the actor effects are presented on the horizontal paths. The estimates of the partner effects are presented on the diagonal paths. Unstandardized path coefficients are shown. ^***^p < 0.001, ^**^p < 0.01, ^*^p < 0.05.

The estimated actor and partner effects are presented in Figure 3.

### Harmonious passion and adaptive outcomes

For the association between HP and PA, evidence was found for the actor effects of both athletes (*b* = 1.520, *z* = 6.465, *p* < 0.001) and coaches (*b* = 1.433, *z* = 7.199, *p* < 0.001) after controlling for the partner effects. Here, athletes and coaches who score higher on HP, report, on average, also higher levels of PA. Further, the partner effect from coaches’ HP to athletes’ PA and from athletes’ HP to coaches’ PA were not statistically significant.

Regarding the association between HP and NS, evidence was again found for the actor effect of both athletes (*b* = 0.379, *z* = 6.372, *p* < 0.001) and coaches (*b* = 0.395, *z* = 7.224, *p* < 0.001) after controlling for the partner effects. In both cases, higher scores on one’s own HP were, on average, associated with higher levels of one’s own NS. Further, evidence was found for the partner effect from athletes to coaches, but here the partner effect was opposite signed in which higher scores on athletes’ HP were associated with lower scores on coaches’ NS (*b* = −0.104, *z* = −1.962, *p* = 0.050).

### Harmonious passion and maladaptive outcomes

Only evidence was found for the athletes’ actor effect (*b* = −0.869, *z* = −3.046, *p* = 0.002), but not for the coaches’ actor effect in the relationship between PA and NA after controlling for the partner effects. Athletes’ who have a higher score on HP, report, on average, lower scores on NA. Further, the athletes’ HP was found to be unrelated to the coaches’ NA, and the coaches’ HP was unrelated to both their own NA and the athletes’ NA.

For the association between HP and NF, again, consistent evidence was found for the two actor effects after controlling for the partner effects. For both athletes (*b* = −0.321, *z* = −5.362, *p* < 0.001) as well as coaches (*b* = −0.210, *z* = −3.071, *p* = 0.002), a negative association was found between their own HP and their own NF experiences. Further, no evidence was found for any partner effect.

### Obsessive passion and adaptive outcomes

Evidence was found for the athlete actor effect (*b* = 0.493, *z* = 3.457, *p* = 0.001) in the association between OP and PA. Therefore, athletes who report a higher score on OP, score higher on PA as well. Further, no evidence was found for the coach actor effect or any of the partner effects.

For the association between OP and NS a similar pattern emerged. Here too, only evidence was found for the athlete actor effect (*b* = 0.082, *z* = 2.226, *p* = 0.026), while no evidence was found for the other three effects.

### Obsessive passion and maladaptive outcomes

With regard to the relationship between OP and NA, evidence was obtained for the actor effects of both athletes (*b* = 0.604, *z* = 3.786, *p* < 0.001) and coaches (*b* = 0.531, *z* = 3.682, *p* < 0.001). As such, coaches and athletes who report higher levels of OP, generally also report higher scores on NA during sports participation or coaching. Further, none of the expected partner effects reached statistical significance.

Finally, for the relationship between OP and NF, only one positive actor effect, namely the one of coaches, was observed (*b* = 0.174, *z* = 4.059, *p* < 0.001). The other actor and partner effects were non-significant, suggesting that OP and NF were, in those cases, not related to each other.

Overall, considering the eight models, only little evidence was found for partner effects. However, prior research has indicated that some partner effects for HP or OP only emerge in more long-term relationships in which dyadic familiarity between the dyad members increases (Jowett et al., 2013). Therefore, in a second stage, the length of the coach-athlete relationship was included as a moderator of both the actor and the partner effects in all eight above-described models. Only one moderation effect, the length of the relationship x the coaches’ harmonious passion partner effect in the association with the athletes’ negative affect, was statistically significant (*b* = −0.003, *z* = −2.044, *p* = 0.041), but the main effect was opposite to the expected direction (*b* = 0.652, *z* = 1.565, *p* = 0.118).

### Actor-partner interdependence mediation models

**Figure 4:**
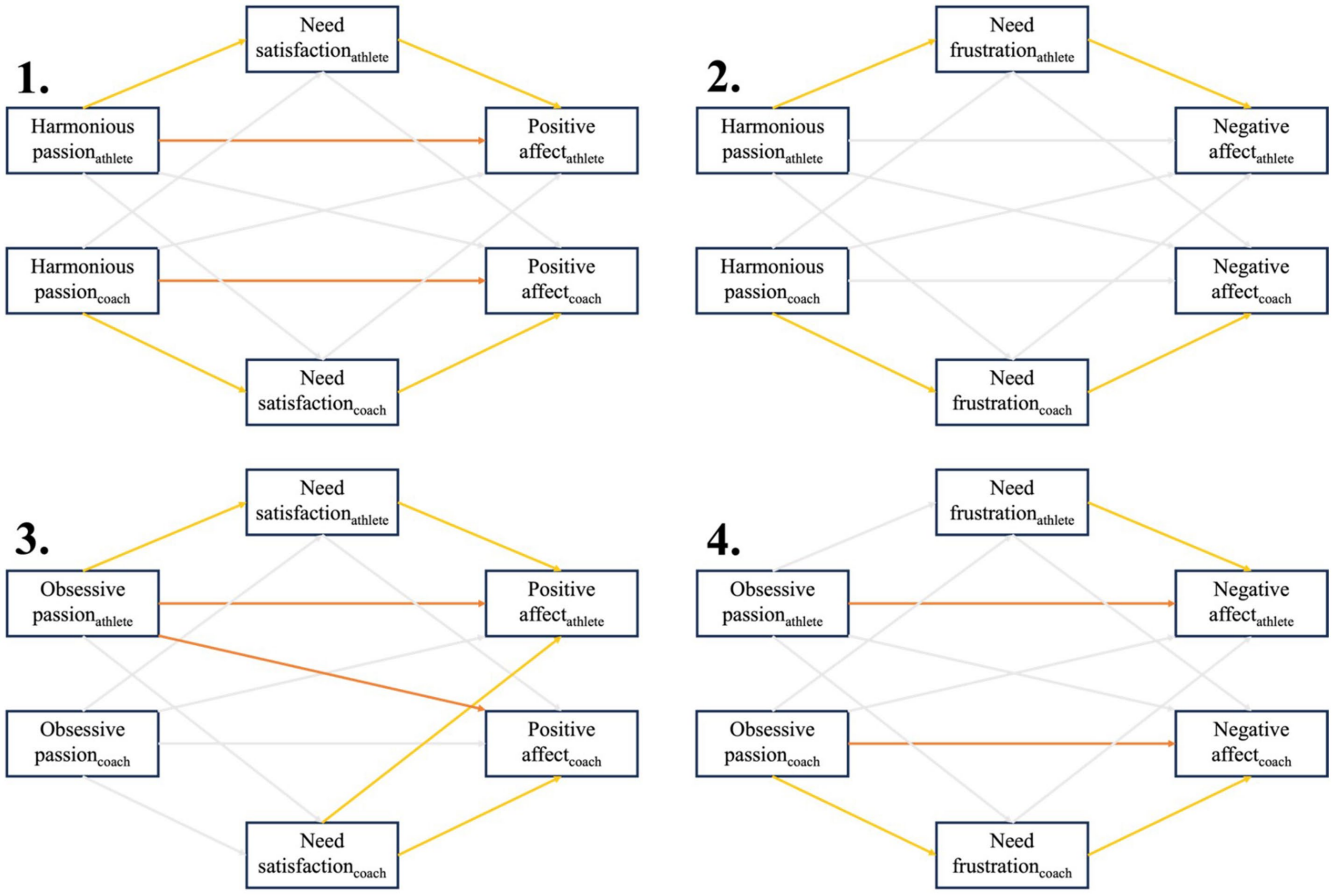
A visual representation of the significant effects of the four actor-partner interdependence mediation models. The yellow arrows represent the significant paths that are part of the indirect effects, while the orange arrow represent the significant direct effects. The gray arrows represent the non-significant paths in each of the models.

Finally, the mediation effects of need satisfaction and frustration were investigated in the relationship between passion and affective experiences. Four Actor-Partner Interdependence Mediation Models were fitted to evaluate the dyadic relationship between (1) harmonious passion and positive affect via need satisfaction; (2) harmonious passion and negative affect via need frustration; (3) obsessive passion and positive affect via need satisfaction; and (4) obsessive passion and negative affect via need frustration. In Figure 4 an overview of the significant path coefficients of the four models is presented. In the Supplementary Tables S3.1–S3.4, the estimates of the total effects, direct effects, total and simple indirect effects as well as the bootstrap percentile 95% confidence intervals are represented. The proportion of the total effect is only reported for the direct and total indirect effect when the total effect is significant.

### The relation between harmonious passion and positive affect via need satisfaction

The total actor effect of HP on PA was significant for both athletes [*b* = 1.520; CI (1.059, 2.012)] and coaches [*b* = 1.433, CI (1.082, 1.788)] (Supplementary Table S3.1). For both the athlete and coach actor effects, evidence was found that this effect was mediated by NS [the total indirect effect for athletes: *b* = 0.347; CI (0.087, 0.623); for coaches: *b* = 0.762; CI (0.497, 1.069)]. For both of them, only the actor-actor indirect effect was statistically significant [for athletes: *b* = 0.294; CI (0.056, 0.531); for coaches: *b* = 0.764; CI (0.499, 1.075)]. This implies that athletes’ and coaches’ scores on HP were positively related to their own NS experiences, which in turn were positively linked to their own PA experiences. Also the direct effect was found to be significant for both athletes [*b* = 1.172; CI (0.630, 1.754)] and coaches [*b* = 0.671; CI (0.224, 1.118)], implying there was only partial mediation. For the athlete actor effect the (total) indirect effect accounted for 23% of the total effect. For the coach actor effect the (total) indirect effect accounted for 53% of the total effect. When disentangling the total athlete and coach partner effect, no further evidence was found for any direct or indirect effect.

### The relation between harmonious passion and negative affect via need frustration

The athlete total actor effect of HP on NA was statistically significant [*b* = −0.869; CI (−1.448, −0.273)] (Supplementary Table S3.2). Evidence was found that this association was mediated by NF experiences [the total indirect effect for athletes: *b* = −0.474; CI (−0.763, −0.210)]. Here, only the actor-actor indirect effect reached statistical significance [*b* = −0.487; CI (−0.770, −0.235)], indicating that athletes who reported a higher score on HP, reported, on average, less NF experiences. Those NF experiences were on its turn related to higher NA experiences. No evidence was found for the athlete actor direct effect. For the athlete actor effect the (total) indirect effect accounted for 55% of the total effect. Despite the absence of evidence for the coach total actor effect, it is still meaningful to examine the decomposition of effects. Here, evidence was found that for coaches the relation between HP and NA was mediated by NF experiences [the total indirect effect: *b* = −0.183; CI (−0.388, −0.031)]. Here too, only the actor-actor indirect effect was evidenced [b = −0.198; CI (−0.401, −0.057)]. Further, when disentangling the total athlete and coach partner effect, no further evidence was found for any direct or indirect effect.

### The relation between obsessive passion and positive affect via need satisfaction

The athlete total actor effect of OP on PA was statistically significant [*b* = 0.493; CI (0.185, 0.763)] (Supplementary Table S3.3). Here, evidence was found for the direct effect of athletes’ OP on their own PA experiences [*b* = 0.416; CI (0.099, 0.718)]. Although, for the athlete actor effect, no evidence was found for the total indirect effect, the actor-actor indirect effect was statistically significant [*b* = 0.102; CI (0.008, 0.220)]. This implies that athletes’ scores on OP were positively related to their own NS experiences, which in turn were positively linked to their own PA experiences. No evidence was found for the partner-partner indirect effect. For the athlete actor effect the (total) indirect effect accounted for 16% of the total effect. When disentangling the coach partner effect, no evidence was found for the total effect and the total indirect effect. However, the direct effect was significant [*b* = −0.260; CI (−0.473, −0.068)]. This implies that athletes’ score on OP is negatively related to the coaches’ PA. When unraveling the total coach actor effect and the total athlete partner effect, no further evidence was found for any direct or indirect effect.

### The relation between obsessive passion and negative affect via need frustration

The total actor effect of OP on NA was significant for both athletes [*b* = 0.604; CI (0.305, 0.936)] and coaches [*b* = 0.531; CI (0.214, 0.846)] (Supplementary Table S3.4). Only for the coach actor effect, evidence was found that this effect was mediated by NF {the total indirect effect for coaches: [*b* = 0.148; CI (0.056; 0.274)]}. Here, only the actor-actor indirect effect was statistically significant [*b* = 0.139; CI (0.048; 0.263)]. This implies that coaches’ scores on OP were positively related to their own NF experiences, which on its turn were positively associated with their own NA experiences. Also the direct effect was found to be significant for the coach actor effect [*b* = 0.383; CI (0.058; 0.717)], implying there was only partial mediation. The total indirect effect accounted for 28% of the total effect in the coach actor effect. For athletes, only the direct effect [*b* = 0.572; CI (0.289, 0.873)] in which athletes’ OP was positively related to their own NA experiences was evidenced.

## Discussion

In this study, we examined the relationship between passion and both more distal affective experiences, and more proximal need-based experiences in coach-athlete dyads. While the existing literature used a unidirectional approach, this study took a dyadic perspective. This allowed to investigate partner effects, in which coaches’ passion affects athletes’ adjustment and vice versa. If this were the case, it would imply that athletes’ affective and need-based experiences are partly influenced by the passion experiences of coaches and vice versa. Furthermore, it allowed to investigate whether the actor effects identified earlier from the actor only perspective, in which implicitly only actor effects were assumed in the unidirectional approach, remain in the less stringent bidirectional approach.

We expected that HP would be positively related to PA and NS experiences and negatively to NA and NF experiences. The study’s results revealed that HP was indeed positively associated with positive affect and need satisfaction, but only at the actor level. However, there was one unexpected, opposite-signed partner effect in which athletes’ HP was negatively associated with coaches’ NS. Further, also the expected negative associations between HP and negative affect and need frustration were confirmed at the actor level. Only the association between coaches’ HP and coaches’ NA was not supported. Here too, no evidence was found for partner effects.

For obsessive passion, we expected a positive association between OP and the less adaptive outcomes of negative affect and need frustration. Again, evidence was found for three out of four actor effects. Only the association between athletes’ OP and athletes’ NA experiences was not evidenced. Again, no evidence was found for partner effects. Prior literature did not reveal a clear picture on the relationship between OP and more adaptive outcomes (Vallerand et al., 2006; Philippe et al., 2009; Stenseng et al., 2015; Verner-Filion et al., 2017; Moen et al., 2018; Lopes and Vallerand, 2020; Schellenberg et al., 2021; Fonteyn and Loeys, 2022). In our study, this was also the case. Only evidence was found for the athlete actor effect in which athletes’ own OP was positively associated with their own NS experiences. However, in all other cases, both at the actor as the partner level, OP was unrelated to NS and PA.

These findings have several important implications. First, the robustness of the actor effects was supported. Our study provided evidence that the two types of passion in athletes and coaches are indeed often (differently) associated with affective experiences and need-based experiences in athletes and coaches respectively, also after controlling for potential partner effects. The few actor effects that were not evidenced in this study align with those that are not consistently found in the existing literature and emphasize a lack of clarity regarding how obsessive passion relates to especially maladaptive outcomes. Secondly, the majority of the partner effects were not supported. This means that coaches’ passion has no impact on athletes’ experiences and vice versa, independent of the type of passion and the more proximal or distal nature of the outcome These findings persisted, even when considering the length of the coach-athlete relationship as a potential moderator.

A potential explanation for the lack of partner effects is that this study only focused on more individual passion outcomes, while the study of Jowett et al. (2013), in which several partner effects were identified, focused on more dyadic outcomes (i.e., relationship satisfaction and interpersonal conflict). It is plausible that the impact of coaches on athletes’ adjustments (and vice versa) limits to those outcomes that are more relational in nature.

The second aim of the present study was to investigate the potential mediation of need-based experiences in the association between the two types of passion and affective experiences. Overall, while for HP evidence was obtained for the intrapersonal indirect effects via need-based experiences in the relationship with both positive and negative affect in both coaches and athletes, for OP only the athletes’ indirect effect in the relationship between OP and PA via NS and the coaches’ indirect effect in the association between OP and NA via NF were supported. This stresses how HP elicits NS experiences and safeguards against NF experiences, which in turn are strongly associated with affect. Similar to the evidence for only actor effects in the basic APIM, also the mediated effects were intrapersonal in nature. Need-based experiences thus form an important intermediate mechanism to understand the impact of passion on more distal passion outcomes. Further research could further explore the conditions or circumstances in which these effects occur most strongly.

### Limitations and future directions

Some important limitations should be acknowledged when interpreting the study’s results. First, this study used a correlational design, making it impossible to draw causal conclusions. Further, this design does not capture the longitudinal nature of the interactions between coaches and athletes. As there is often an intensive cooperation between both, some more fine-grained processes and interactions were not captured by the use of the cross-sectional design. Also, coaches and athletes were both experienced in our sample. Effects in inexperienced athletes and coaches need to be verified, as well as how interaction over time evolves. Therefore, future research should consider a longitudinal design in which trait and occasion-specific processes can be differentiated.

Second, part of the data collection took place during the COVID-19 pandemic. The impact of the pandemic might have been greater than anticipated, especially when it comes to the partner effects. Certain restrictions or measures in specific sports disciplines may have led, for instance, to fewer or different interactions between coaches and athletes. Nevertheless, our findings align with prior research that explicitly examined the impact of COVID on passion experiences in individual sport experiences, revealing predominant evidence for actor effects and only limited support for partner effects.

Third, incorporating need frustration as a proximal passion outcome and as a potential mediator variable addresses an existing gap in the literature. However, the reliability of this measurement was relatively low, particularly for athletes. Consequently, these results should be interpreted with caution. While we only presented aggregate scales for both NS and NF in this paper, all analyses were conducted for each of the three need subscales separately as well. One might argue for example that partner effects will be more pronounced for the relatedness component. Nonetheless, the findings from these more fine-grained analyses (results not shown) were comparable to those presented here. It is important to note, however, that both satisfaction and frustration of each subscale were assessed using only two items per need. Therefore, future research could benefit from using a broader range of items for the measurement of each of the needs to assess whether different patterns emerge for each of the needs.

Fourth, all constructs were measured by self-reports of athletes and coaches. Although these reports are relevant, they are also considered more subjective measures. Therefore, it might be important to incorporate more objective assessments in future research.

Fifth, other intermediate mechanisms could play a role in how coaches’ passion experiences impact athletes’ adjustment (and vice versa) as well. For example, the coaching style used by the coach may be a significant factor when considering athletes’ experiences (Lafrenière et al., 2011). However, preliminary analysis in this study (results not shown), did not reveal any evidence for this. Further research, incorporating more comprehensive assessments of the interpersonal style used in the sports context, could provide greater insight into this matter.

Finally, given that this study did not provide support for the identification of partner effects, it raises the question of whether a dyadic perspective is truly required. However, different contexts may give rise to varying underlying dyadic patterns. In our study, the actor only pattern appears to be predominant, but different dyadic configurations may emerge in other settings. We refrain from generalizing, and researchers are encouraged to employ a dyadic approach to study dynamics between athletes and coaches. It is still possible that one’s passion may have an impact on other important constructs that determine one’s partner’s sport experiences but that were not included in this study.

## Conclusion

This study aimed to address several important limitations on the role of passion in the sports literature. While prior studies mostly used a unidirectional approach, this study used a dyadic approach. Despite the lack of substantial evidence for partner effects, our study did support the robustness of most actor effects. Further, some of these associations were previously only investigated in athletes and are now showed in coaches as well (or vice versa). Our study confirmed prior evidence for how the two types of passion are associated with distinct outcomes. In both athletes and coaches HP is consistently and positively linked to more adaptive outcomes and negatively to less adaptive outcomes, whereas OP exhibits a negative association with these less adaptive outcomes. As all the evidenced associations are confined to the intrapersonal level, being harmoniously passionate seems to primarily function as a protective factor for the individuals themselves, rather than extending to the person who are in a close relationship with them. Conversely, the risks associated with being obsessively passionate also appear to be constrained to the individual who is obsessively passionate without extending the impact of OP to the close relationship partner. Our study also supported some first evidence for the mediating role of need-based experiences in the relationship between passion and more distal affective outcomes and gave a first deeper understanding of the underlying mechanisms through which passion impacts one’s affective experiences in sports.

## Data availability statement

The raw data supporting the conclusions of this article will be made available by the authors, without undue reservation.

## Ethics statement

The studies involving humans were approved by Ethics Committee Faculty of Psychology and Educational Sciences of Ghent University. The studies were conducted in accordance with the local legislation and institutional requirements. Written informed consent for participation in this study was provided by the participants’ legal guardians/next of kin.

## Author contributions

MF: Conceptualization, Data curation, Formal analysis, Methodology, Project administration, Visualization, Writing – original draft, Writing – review & editing. LH: Writing – original draft, Writing – review & editing. MV: Writing – original draft, Writing – review & editing. TL: Conceptualization, Methodology, Writing – original draft, Writing – review & editing.
