# Supplementary material for: Is passion contagious in coach-athlete dyads? A dyadic exploration of the association between passion, affective and need-based experiences in individual sports
Source: Front Psychol. 2024 Apr 24;15:1369011. doi: 10.3389/fpsyg.2024.1369011 (PMC11077636; doi:10.3389/fpsyg.2024.1369011)
Supplement: Supplementary file 1 [file Table_1.DOCX]

**Table S1**

*Table S1. Reliabilities Measurements*

| Variable | Cronbach Alpha  (95% CI) | Alpha if Item Deleted  (min-max) |
| --- | --- | --- |
| Harmonious Passion Athlete | 0.63 (0.54,0.70) | 0.54-0.61 |
| Harmonious Passion Coach | 0.68 (0.61,0.74) | 0.62-0.66 |
| Obsessive Passion Athlete | 0.82 (0.78,0.86) | 0.77-0.81 |
| Obsessive Passion Coach | 0.82 (0.78,0.86) | 0.78-0.81 |
| Positive Affect Athlete | 0.75 (0.69,0.80) | 0.67-0.75 |
| Positive Affect Coach | 0.79 (0.74,0.83) | 0.72-0.78 |
| Negative Affect Athlete | 0.72 (0.66,0.78) | 0.59-0.75 |
| Negative Affect Coach | 0.60 (0.51,0.69) | 0.39-0.62 |
| Need Satisfaction Athlete | 0.69 (0.61,0.75) | 0.59-0.70 |
| Need Satisfaction Coach | 0.73 (0.67,0.79) | 0.67-0.73 |
| Need Frustration Athlete | 0.47 (0.35,0.58) | 0.39-0.51 |
| Need Frustration Coach | 0.61 (0.52,0.69) | 0.53-0.61 |

**Table S2**

*Table S2. An overview of the Effects, Coefficients and Corresponding Labels of the Different APIMeM Parameters.*

| Effect | Coefficient | | | Label |
| --- | --- | --- | --- | --- |
| Athlete actor effect |  |  |  |  |
| Total effect | a_AA_b_AA_ + a_PC_b_PA_ + c’_AA_ | |  | Athlete actor total effect |
| Total IE | a_AA_b_AA_ + a_PC_b_PA_ | |  | Athlete actor total indirect effect |
| Actor-actor IE | a_AA_b_AA_ | |  | Athlete actor-actor indirect effect |
| Partner-partner IE | a_PC_b_PA_ | |  | Athlete partner-partner indirect effect |
| Direct effect | c’_AA_ | |  | Athlete actor direct effect |
| Coach actor effect |  |  |  |  |
| Total effect | a_AC_b_AC_ + a_PA_b_PC_ + c’_AC_ | |  | Coach actor total effect |
| Total IE | a_AC_b_AC_ + a_PA_b_PC_ | |  | Coach actor total indirect effect |
| Actor-actor IE | a_AC_b_AC_ | |  | Coach actor-actor indirect effect |
| Partner-partner IE | a_PA_b_PC_ | |  | Coach partner-partner indirect effect |
| Direct effect | c’_AC_ | |  | Coach actor direct effect |
| Athlete partner effect |  |  |  |  |
| Total effect | a_AC_b_PA_ + a_PA_b_AA_ + c’_PA_ | |  | Athlete partner total effect |
| Total IE | a_AC_b_PA_ + a_PA_b_AA_ | |  | Athlete partner total indirect effect |
| Actor-partner IE | a_AC_b_PA_ | |  | Athlete actor-partner indirect effect |
| Partner-actor IE | a_PA_b_AA_ | |  | Athlete partner-actor indirect effect |
| Direct effect | c’_PA_ | |  | Athlete partner direct effect |
| Coach partner effect |  |  |  |  |
| Total effect | a_AA_b_PC_ + a_PC_b_AC_ + c’_PC_ | |  | Coach partner total effect |
| Total IE | a_AA_b_PC_ + a_PC_b_AC_ | |  | Coach partner total indirect effect |
| Actor-partner IE | a_AA_b_PC_ | |  | Coach actor-partner indirect effect |
| Partner-actor IE | a_PC_b_AC_ | |  | Coach partner-actor indirect effect |
| Direct effect | c’_PC_ | |  | Coach partner direct effect |

**Tables S3**

*Table S3.1. Total, Direct and Indirect Effects and the Corresponding Bootstrap Percentile 95% Confidence Intervals (CI) for the Effect of Harmonious Passion on Positive Affect via Need Satisfaction*

| Effect | Estimate | 95% CI | | Proportion of the total effect |
| --- | --- | --- | --- | --- |
|  |  | Lower | Upper |  |
| Athlete actor effect |  |  |  |  |
| Total effect | 1.520 | 1.059 | 2.012 |  |
| Total IE | 0.347 | 0.087 | 0.623 | 22.83% |
| Actor-actor IE | 0.294 | 0.056 | 0.531 |  |
| Partner-partner IE | 0.054 | -0.009 | 0.155 |  |
| Direct effect | 1.172 | 0.630 | 1.754 | 77.11% |
| Coach actor effect |  |  |  |  |
| Total effect | 1.433 | 1.082 | 1.788 |  |
| Total IE | 0.762 | 0.497 | 1.069 | 53.18% |
| Actor-actor IE | 0.764 | 0.499 | 1.075 |  |
| Partner-partner IE | -0.003 | -0.041 | 0.031 |  |
| Direct effect | 0.671 | 0.224 | 1.118 | 46.82% |
| Athlete partner effect |  |  |  |  |
| Total effect | 0.140 | -0.312 | 0.642 |  |
| Total IE | -0.154 | -0.401 | 0.096 |  |
| Actor-partner IE | -0.204 | -0.444 | 0.024 |  |
| Partner-actor IE | 0.050 | -0.024 | 0.164 |  |
| Direct effect | 0.294 | -0.209 | 0.838 |  |
| Coach partner effect |  |  |  |  |
| Total effect | -0.176 | -0.606 | 0.217 |  |
| Total IE | -0.216 | -0.549 | 0.034 |  |
| Actor-partner IE | -0.015 | -0.170 | 0.138 |  |
| Partner-actor IE | -0.201 | -0.461 | 0.000 |  |
| Direct effect | 0.040 | -0.367 | 0.419 |  |

*Table S3.2. Total, Direct and Indirect Effects and the Corresponding Bootstrap Percentile 95% Confidence Intervals (CI) for the Effect of Harmonious Passion on Negative Affect via Need Frustration*

| Effect | Estimate | 95% CI | | Proportion of the total effect |
| --- | --- | --- | --- | --- |
|  |  | Lower | Upper |  |
| Athlete actor effect |  |  |  |  |
| Total effect | -0.869 | -1.448 | -0.273 |  |
| Total IE | -0.474 | -0.763 | -0.210 | 54.55% |
| Actor-actor IE | -0.487 | -0.770 | -0.235 |  |
| Partner-partner IE | 0.012 | -0.061 | 0.098 |  |
| Direct effect | -0.395 | -0.982 | 0.146 | 45.45% |
| Coach actor effect |  |  |  |  |
| Total effect | -0.353 | -0.772 | 0.070 |  |
| Total IE | -0.183 | -0.388 | -0.031 |  |
| Actor-actor IE | -0.198 | -0.401 | -0.057 |  |
| Partner-partner IE | 0.015 | -0.025 | 0.074 |  |
| Direct effect | -0.170 | -0.613 | 0.269 |  |
| Athlete partner effect |  |  |  |  |
| Total effect | 0.001 | -0.635 | 0.554 |  |
| Total IE | -0.170 | -0.432 | 0.053 |  |
| Actor-partner IE | -0.089 | -0.240 | 0.024 |  |
| Partner-actor IE | -0.081 | -0.311 | 0.093 |  |
| Direct effect | 0.171 | -0.411 | 0.717 |  |
| Coach partner effect |  |  |  |  |
| Total effect | 0.254 | -0.083 | 0.757 |  |
| Total IE | 0.118 | -0.104 | 0.348 |  |
| Actor-partner IE | 0.090 | -0.047 | 0.259 |  |
| Partner-actor IE | 0.027 | -0.114 | 0.158 |  |
| Direct effect | 0.136 | -0.361 | 0.637 |  |

*Table S3.3. Total, Direct and Indirect Effects and the Corresponding Bootstrap Percentile 95% Confidence Intervals (CI) for the Effect of Obsessive Passion on Positive Affect via Need Satisfaction*

| Obsessive passion – need satisfaction – positive affect | | | | |
| --- | --- | --- | --- | --- |
| Effect | Estimate | 95% CI | | Proportion of the total effect |
|  |  | Lower | Upper |  |
| Athlete actor effect |  |  |  |  |
| Total effect | 0.493 | 0.185 | 0.763 |  |
| Total IE | 0.077 | -0.026 | 0.183 | 15.62% |
| Actor-actor IE | 0.102 | 0.008 | 0.220 |  |
| Partner-partner IE | -0.025 | -0.084 | 0.015 |  |
| Direct effect | 0.416 | 0.099 | 0.718 | 84.38% |
| Coach actor effect |  |  |  |  |
| Total effect | 0.149 | -0.178 | 0.437 |  |
| Total IE | -0.013 | -0.236 | 0.156 |  |
| Actor-actor IE | -0.008 | -0.228 | 0.160 |  |
| Partner-partner IE | -0.005 | -0.034 | 0.013 |  |
| Direct effect | 0.162 | -0.075 | 0.383 |  |
| Athlete partner effect |  |  |  |  |
| Total effect | 0.010 | -0.281 | 0.298 |  |
| Total IE | -0.045 | -0.148 | 0.064 |  |
| Actor-partner IE | 0.002 | -0.043 | 0.063 |  |
| Partner-actor IE | -0.047 | -0.148 | 0.048 |  |
| Direct effect | 0.055 | -0.210 | 0.327 |  |
| Coach partner effect |  |  |  |  |
| Total effect | -0.141 | -0.411 | 0.109 |  |
| Total IE | 0.120 | -0.050 | 0.309 |  |
| Actor-partner IE | 0.011 | -0.019 | 0.056 |  |
| Partner-actor IE | 0.109 | -0.056 | 0.298 |  |
| Direct effect | -0.260 | -0.473 | -0.068 |  |

*Table S3.4. Total, Direct and Indirect Effects and the Corresponding Bootstrap Percentile 95% Confidence Intervals (CI) for the Effect of Obsessive Passion on Negattive Affect via Need Frustration*

| Obsessive passion – need frustration – negative affect | | | | |
| --- | --- | --- | --- | --- |
| Effect | Estimate | 95% CI | | Proportion of the total effect |
|  |  | Lower | Upper |  |
| Athlete actor effect |  |  |  |  |
| Total effect | 0.604 | 0.305 | 0.936 |  |
| Total IE | 0.032 | -0.103 | 0.175 | 5.30% |
| Actor-actor IE | 0.035 | -0.086 | 0.174 |  |
| Partner-partner IE | -0.002 | -0.045 | 0.038 |  |
| Direct effect | 0.572 | 0.289 | 0.873 | 94.70% |
| Coach actor effect |  |  |  |  |
| Total effect | 0.531 | 0.214 | 0.846 |  |
| Total IE | 0.148 | 0.056 | 0.274 | 27.87% |
| Actor-actor IE | 0.139 | 0.048 | 0.263 |  |
| Partner-partner IE | 0.009 | -0.015 | 0.053 |  |
| Direct effect | 0.383 | 0.058 | 0.717 | 72.13% |
| Athlete partner effect |  |  |  |  |
| Total effect | -0.089 | -0.442 | 0.236 |  |
| Total IE | 0.012 | -0.165 | 0.197 |  |
| Actor-partner IE | 0.064 | -0.026 | 0.182 |  |
| Partner-actor IE | -0.052 | -0.186 | 0.069 |  |
| Direct effect | -0.101 | -0.425 | 0.213 |  |
| Coach partner effect |  |  |  |  |
| Total effect | 0.025 | -0.244 | 0.319 |  |
| Total IE | -0.011 | -0.078 | 0.060 |  |
| Actor-partner IE | -0.006 | -0.039 | 0.022 |  |
| Partner-actor IE | -0.005 | -0.073 | 0.062 |  |
| Direct effect | 0.036 | -0.234 | 0.308 |  |
